# Efficacy of tibial cortex transverse transport in treating diabetic foot ulcer and its effect on serum omentin-1 and irisin levels

**DOI:** 10.1186/s13098-024-01400-1

**Published:** 2024-07-09

**Authors:** Yang Wen, Liyuan Chen, Jiaping Lan, Lei Li

**Affiliations:** 1 Orthopedic Center, Orthopaedic Trauma, Suining Central Hospital, No. 27 Dongping North Road, Hedong New District, Suining, 629000 Sichuan China; 2 Medical Department, Suining Central Hospital, Suining, 629000 Sichuan China

**Keywords:** Diabetic foot ulcer, Tibial cortex transverse transport, VEGF, Omentin-1, Irisin

## Abstract

**Objective:**

Diabetic foot ulcer (DFU) is a common and debilitating complication of diabetes that is associated with an increased risk of lower-limb amputation and a reduced life expectancy. Tibial cortex transverse transport (TTT) has become a newly alternative surgical method to facilitate ulcer healing and prevent lower limb amputation. Herein, we investigated the efficacy of TTT in treating DFU and changes of serum omentin-1 and irisin levels.

**Methods:**

This study prospectively recruited 52 consecutive patients with DFU who were treated with TTT. The follow-up was performed weekly during the first 12 weeks postoperatively and every 3 months until 1 year after TTT. The serum levels of vascular endothelial growth factor (VEGF), omentin-1, and irisin in DFU patients undergoing TTT were determined by ELISA methods on the preoperative 1st day, postoperative 2nd week and 4th week.

**Results:**

The wound healing rate was 92.3% (48/52) at the 1-year follow-up. The visual analog scale (VAS) pain scores of patients showed a significant reduction at the 4th week after TTT (*p* < 0.001). The dorsal foot skin temperature, ankle brachial index, and dorsal foot blood flow of patients were significantly increased at the 4th week after TTT (*p* < 0.001). Results of ELISA methods showed the serum levels of VEGF, omentin-1, and irisin on the 2nd week and 4th week after TTT were notably elevated compared to the levels determined on the preoperative 1st day (*p* < 0.001). The serum levels of VEGF, omentin-1, and irisin on the 4th week after TTT were also significantly higher than the levels determined on the 2nd week after TTT (*p* < 0.001).

**Conclusion:**

TTT could promote the wound healing and reduce the risk of lower limb amputation, demonstrating promising clinical benefits in the treatment of DFU. Increased expressions of serum proangiogenic factors including VEGF, omentin-1, and irisin were noted in the early stage after TTT, which may provide a new mechanism of TTT promoting wound heal.

## Introduction

Diabetic foot ulcers (DFUs) affect nearly 18.6 million people worldwide each year, which remain a major source of infection, hospitalization, non-traumatic lower limb amputations in adults with diabetes, leading to significant morbidity and mortality [1]. The etiology of DFUs is complex because of their multifactorial nature involving diabetic neuropathy, peripheral vascular disease, and hyperglycemia alone or together [2]. The recurrence rate at 3–5 years of incident ulceration is 65%, the incidence rate of lifetime lower-extremity amputation is 20%, and the 5-year mortality is 50–70% [3]. Over the past 25 years, the therapeutic modalities of DFU have significantly advanced and established, including surgical therapies, such as debridement, revascularization, and microsurgical reconstruction using flaps, as well as non-surgical therapies, such as mechanical offloading and negative pressure wound therapy [4]. Recent advances in adjunctive therapies with noninvasive characterization for DFU focus on bioactive dressings, mesenchymal stem cell-based therapy, and platelet and cytokine-based therapy [5]. Although patients with mild-to-moderate ulcers can achieve clinical outcomes from these therapies, those with severe or recalcitrant ulcers are still confront with minor response to therapy, high complication, and major and minor lower limb amputations [6, 7]. A joint group of Chinese Medical Association (CMA) and Chinese Medical Doctor Association (CMDA) expert representatives have reached a consensus that the treatment of lower extremity vasculopathy is the focus of clinical practice to promote the healing process of DFUs [8].

Microcirculation impairment has been implicated as a cause of poor wound healing and worse outcomes commonly observed in DFUs [9]. Sustained slow distraction of osteotomized bone segments with the Ilizarov techniques give the skeleton a suitable stretch stress, which can activate new and old tissue metabolism and stimulate angiogenesis in the bone itself and the surrounding tissues [10]. Tibial cortex transverse transport (TTT) is a novel surgical method based on the Ilizarov tension-stress rule, which has received much attention for its primary function to rebuild microcirculation without changing limb length, relieve ischemic symptoms, and promote wound healing in DFUs [11]. Previous studies provided the results that this technique promoted ulcer healing, facilitated limb salvage, and increased perfusion at the foot in patients with severe or recalcitrant DFUs compared to established surgical therapies [12, 13]. In addition to clinical trials, recently accumulated preclinical models have emerged to explore the molecular mechanism behind enhanced angiogenesis and bone tissue formation after TTT [14]. Omentin-1 is an adipokine with anti-inflammatory properties and exerting proangiogenic functions, and its abnormally reduced expression are associated with disease severity in patients with diabetes and peripheral artery disease or DFUs [15]. Irisin is an emerging adipokine that can promote fracture healing by improving osteogenesis and angiogenesis [16], and its low circulating level is associated with chronic complications in the context of diabetes [17, 18]. In this study, we sought to investigate the efficacy of TTT in treating DFU and the effects of this technique on serum omentin-1 and irisin levels.

## Methods

### Patient selection

This study prospectively recruited consecutive patients with DFU who were treated with TTT. This recruitment occurred from January 2020 to December 2022 and approved by the Ethics Committee of our hospital. The inclusion criteria were (i) patients with a diagnosis of diabetes by the American Diabetes Association criteria [19]; (ii) non-healing or recurrent ulcers (2-B to 3-D grades based on University of Texas wound classification system [20]) in the lower extremities for at least 2 months; (iii) patients failing to respond to previous non-surgical treatments (e.g., wound care and negative pressure wound therapy) and previous surgical management (e.g., serial debridement, revascularization, and microsurgical reconstruction using flaps or skin graft) for at least 8 weeks; (iv) patients aged ≥ 18 years; and (v) patients receiving 1-year follow-up. The exclusion criteria were (i) ulcers extending above the ankle; (ii) a malignant disease in the ulcers; (iii) ulcers not related to diabetes; (iv) infection in the surgical area of the calf; (v) active Charcot’s arthropathy of the foot; or (vi) acute critical limb ischemia (occlusion ≥ 80% of the lumen) and unable to receive vascular reconstruction.

### Surgical techniques

**Fig. 1:**
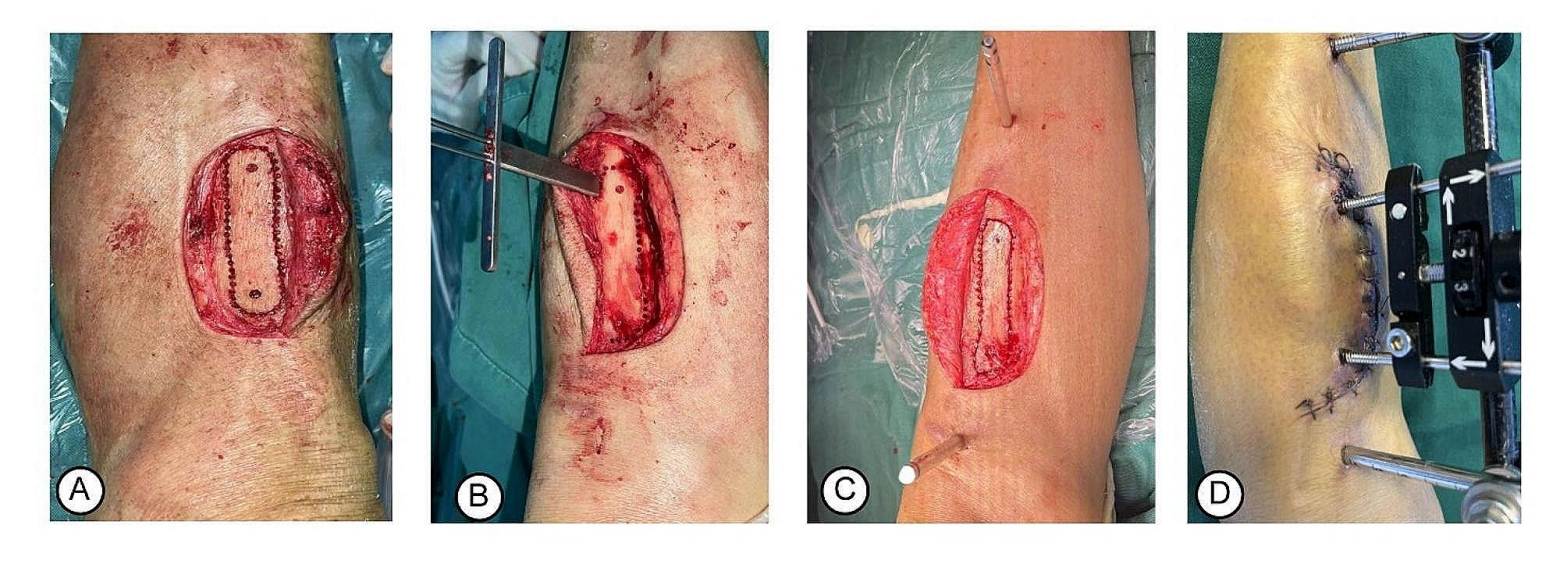
Intraoperative photograph for TTT procedures. (**A**) The corticotomy window (5 cm × 1.5 cm); (**B**) The corticotomy was performed using multiple unicortical drill holes; (**C**) Two nails into the tibia for fixation of the external frame; (**D**) The components are assembled into a completed bone distraction device

Patients were given TTT along the anteromedial part of the proximal tibia in the affected limb under spinal anesthesia or femoral nerve blockage by a senior orthopaedic surgeon. In brief, an arc skin incision (6 cm to 8 cm) was made along the anteromedial part of the proximal tibia. After drilling holes one by one along the rectangle on the tibial cortex, the corticotomy was performed in a vertical rectangle (5 cm × 1.5 cm) which was also known as a corticotomy window (Fig. 1A). After osteotomy, two fixed nails (4 mm diameter for each) were inserted into the corticotomy window for distraction, and two external fixators (5 mm diameter for each) were parallelly installed into both the distal and proximal ends of the tibia to form a stable construct for tibial cortex transport (Fig. 1B-D). The periosteum was sewn layer by layer, followed by the suture of subcutaneous tissue and skin. The operation was finished after bandaging the incision.

### Postoperative management

After operation, patients were required to strictly control their blood sugar, blood lipids, blood pressure, avoid infection, correct hypoproteinemia and electrolyte disturbances. The affected limb was appropriately elevated to reduce swelling. On postoperative day 1, X-rays was performed on the lateral side of the affected tibia. TTT is an invasive operation that can activate the immune system, and patients may experience symptoms such as fever, itching, rash, swelling, and pain. Four-day latency before transport was set to avoid infection and for better tolerance to transport. Following a 4-day latency period, TTT was initiated and adjusted by 1 mm per day (four times with 0.25 mm per time). The period of TTT consisted of 2 weeks of medial transport and 2 weeks of lateral transport. Afterwards, the corticotomy window was reset followed by capture of X-ray images. If no abnormal findings were observed on the X-ray images, the external fixator was removed.

### Outcome measurements

The follow-up was performed weekly during the first 12 weeks postoperatively and every 3 months until 1 year after TTT. The wound healing rate was determined at 1 year after surgery. A healed wound was defined as complete epithelialization without drainage of a previous ulcer site and lasting for 2 weeks [21]. Recurrence was defined as an occurrence of new ulcers, irrespective of location and time, since previous ulcer. The visual analog scale (VAS) pain score ranging 1 to 10, skin temperature between 09.00 h and 12.00 h and between 14.00 h and 17.00 h, ankle brachial index (ABI), and blood flow (ml/100 g/min) were used to evaluate clinical outcomes.

### Blood collection and enzyme linked immunosorbent assay (ELISA) methods

The peripheral blood samples were collected from fasting patients on the preoperative 1st day, postoperative 2nd week and 4th week, and then placed into pyrogen/endotoxin-free tubes. The serum was separated by centrifugation at 3000 × g for 10 min. The serum samples were allowed to be tested by commercially available human ELISA kits for VEGF (DVE00, R&D Systems, Minneapolis, MN, USA), omentin-1 (Cat. DY4254-05, R&D Systems), and irisin (Cat. DY9420-05, R&D Systems) with the instructions of manufacturers. Briefly, the Assay Diluent (a buffered protein base) was added to the microplate (100 µl per well), followed by the addition of VEGF, omentin-1, and irisin standards or samples (100 µl per well). The microplates were sealed and incubated for 2 h at room temperature, followed by three times of washes (400 µl wash buffer per well) The detection reagent, a peroxidase-conjugated anti-VEGF antibody, anti-omentin-1 antibody, or anti-irisin antibody was added to the microplates (200 µl per well). The microplates were incubated for 2 h at room temperature, followed by three times of washes (400 µl wash buffer per well) and the addition of peroxidase-specific substrate (200 µl per well). After 20 min, the peroxidase reaction was terminated by adding Stop solution (2 N sulfuric acid) into the microplates (50 µl per well). The color intensity was proportional to the concentration of VEGF, omentin-1, and irisin.

### Statistical analysis

Results in this study were summarized as a mean with standard deviation (s.d.), and a median with range (quartile 1, quartile 3). Difference for results from the 1st day before surgery to different time points after surgery after was determined by using paired t test, Wilcoxon matched-pairs signed rank test, or the one-way analysis of variance (ANOVA) followed by Tukey’s post hoc test. Statistical analysis and result visualization were carried out by using GraphPad Prism 8 (GraphPad Software, USA) and the possibility less than 0.05, shown as *P* < 0.05, indicates a significant difference.

## Results

### Demographics of the patients

**Fig. 2:**
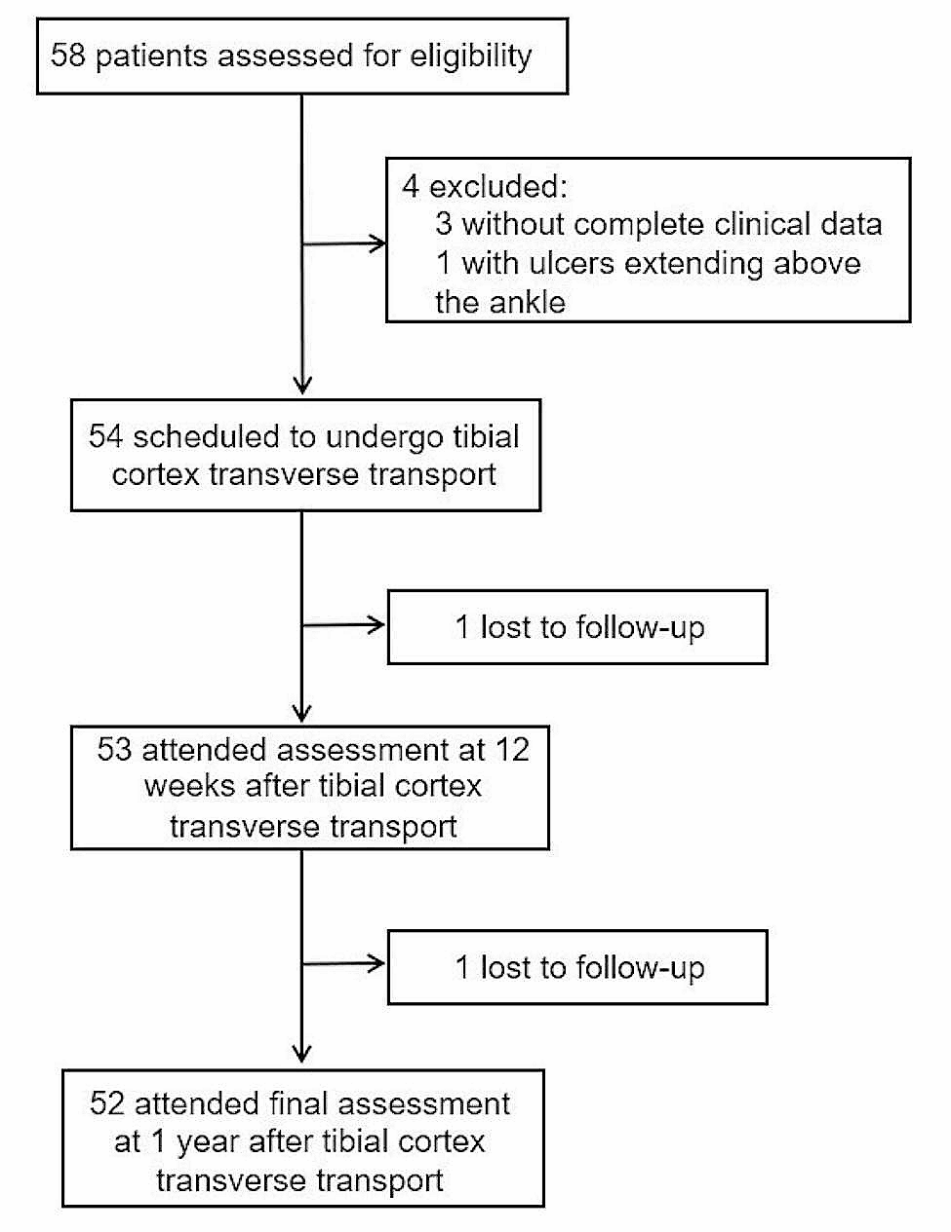
The study flowchart

During the recruitment period, a total of 58 patients were evaluated for eligibility, with 4 patients failing at initial screening. Resulting 54 patients received TTT and were followed up. Due to 2 being lost to follow up, a final 52 patients were eligible for the observation (Fig. 2). The entire cohort was composed of 20 women and 32 men, with age ranging from 48 to 82 years. Among 52 patients, 27 patients had TTT on their left feet and 25 patients had TTT on their right feet. According to university of Texas wound classification system, there were 9 patients graded as 2B, 5 patients as 2 C, 11 patients as 2D, 10 patients as 3B, 2 patients as 3 C, and 14 patients as 3D.

### Wound healing after TTT

**Fig. 3:**
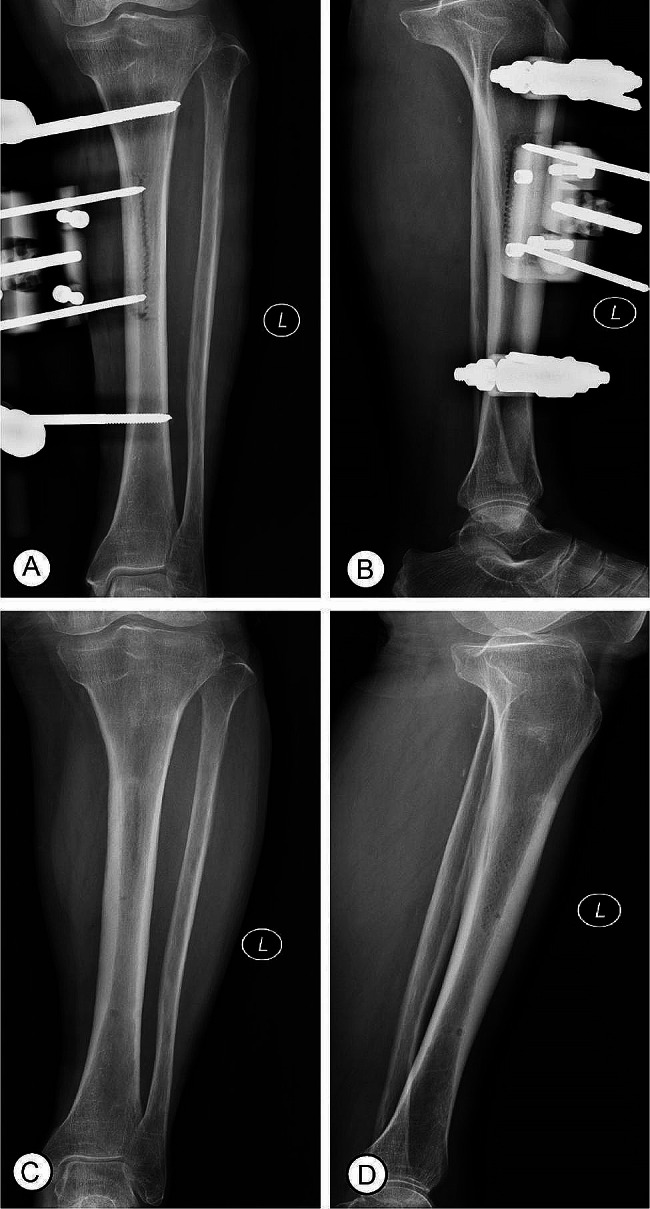
X-ray films of the osteotomy area. (**A**) The corticotomy and external fixation frame; (**B**) The lateral bone movement on the 1st day after TTT; (**C-D**) Tibial osteotomy area heals well following external fixator removal

**Fig. 4:**
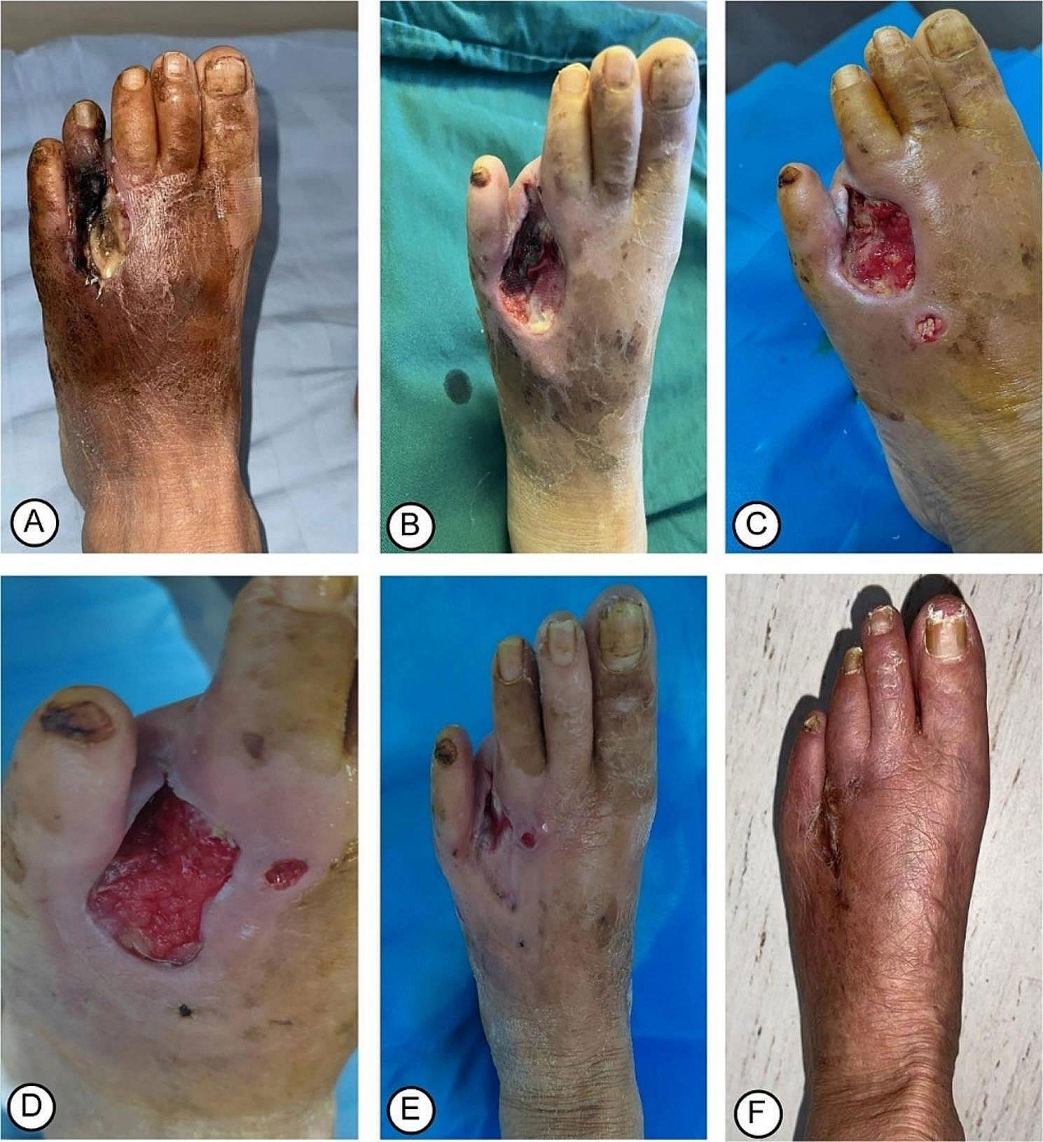
Photograph showing the wound healing process of a 55-year-old woman with DFU. (**A**) Before debridement, the wound failed to heal, covered by necrotic tissues and purulent discharge; (**B**) The fourth toe had been amputated and the necrotic tissues were removed after debridement. (**C-D**) Two weeks after TTT, the red granulation tissue gradually growing on the ulcer. (**E**) Four weeks after TTT, the wound was much narrower with epithelization at the edges. (**F**) Six weeks after TTT, the wound was completely healed

**Fig. 5:**
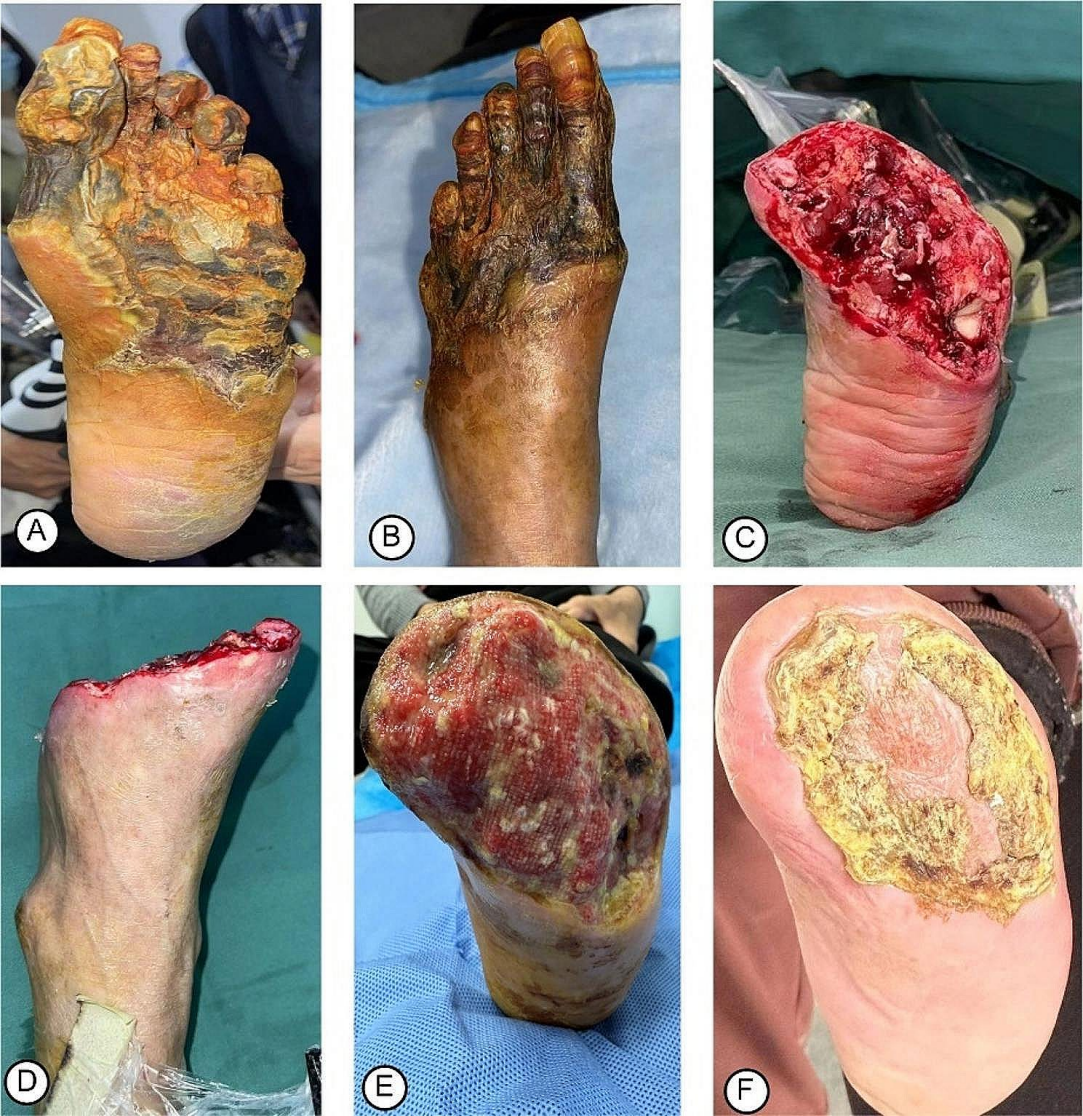
Photograph showing the wound healing process of a 58-year-old man with severe DFUs. (**A-B**) The forefoot was partially necrotic before TTT; (**C-D**) The forefoot had been amputated followed by debridement, with necrotic tissues removed and leaving a huge wound. (**E**) Four weeks after TTT, the wound was covered by robust, fresh granulation tissue; (**F**) Twelve weeks after TTT, the ulcer was completely healed

An X-ray film of the osteotomy area showed position of the corticotomy and external fixation frame (Fig. 3A), as well as lateral bone movement on the 1st day after TTT (Fig. 3B). X-ray films after a 2-week lateral transport showed bone healing following external fixator removal (Fig. 3C, D). In most patients (Fig. 4A, B), there was red granulation tissue gradually growing on the ulcers on the 2nd week after TTT (Fig. 4C, D), and there were signs of healing as shown by epithelialization of the ulcer surface at the 4th week after TTT (Fig. 4E). Figure 3F shows the ulcers were completely healed at the 6th week after TTT. In patients with severe DFUs (Fig. 5A-D), the wound was covered by robust, fresh granulation tissue at the 4th week after TTT (Fig. 5E) and the ulcer was completely healed at the 12th week after TTT (Fig. 5F). The wound healing time was 11.8 ± 3.2 weeks, ranging from 5 to 15 weeks. The mean value of wound area was 8.5 cm^2^ before TTT and 3.7 cm^2^ at the 4th week after TTT, showing a significant reduction (Table 1, *p* < 0.001). The wound healing rate was 92.3% (48/52) at the 1-year follow-up. At the 1-year follow-up, 1 patient (1.9%) underwent amputation and 2 patients (3.8%) experienced recurrence.

**Table 1:** The wound healing area, VAS scores, dorsal foot skin temperature, ABI, and dorsal foot blood flow of patients before TTT and 1 at the 4th week after TTT

Item	Before	Postoperative 4 weeks	*p*
Wound area (cm^2^, mean ± s.d.)	8.5 ± 2.6	3.7 ± 1.3	< 0.001^#^
VAS score [median, (Q1, Q3)]	5 (5, 5)	1 (1, 2)	< 0.001^*^
Dorsal foot skin temperature (°C)	28.6 ± 0.7	31.2 ± 0.5	< 0.001^#^
ABI	0.55 ± 0.10	0.75 ± 0.09	< 0.001^#^
Dorsal foot blood flow (ml/100 g/min)	13.4 ± 3.4	27.6 ± 4.1	< 0.001^#^

VAS: visual analog scale; ABI: ankle brachial index; ^#^ indicates *p* value yielded by paired t test; ^*^ indicates *p* value yielded by Wilcoxon matched-pairs signed rank test

### Postoperative pain and foot conditions after TTT

The median value of VAS scores was 5 before TTT and 1 at the 4th week after TTT, showing a significant reduction (*p* < 0.001). The mean value of dorsal foot skin temperature was 28.6 °C before TTT and 31.2 °C at the 4th week after TTT, indicating a remarkable elevation (*p* < 0.001). The ABI of patients was significantly increased at 4 weeks after TTT, with a mean value from preoperative 0.55 to postoperative 0.75 (*p* < 0.001). The dorsal foot blood flow of patients was significantly increased at 4 weeks after TTT, with a mean value from preoperative 13.4 ml/100 g/min to postoperative 27.6 ml/100 g/min (*p* < 0.001). All data are presented in Table 1.

### Serum levels of VEGF, omentin-1, and irisin in DFU patients after TTT

**Fig. 6:**
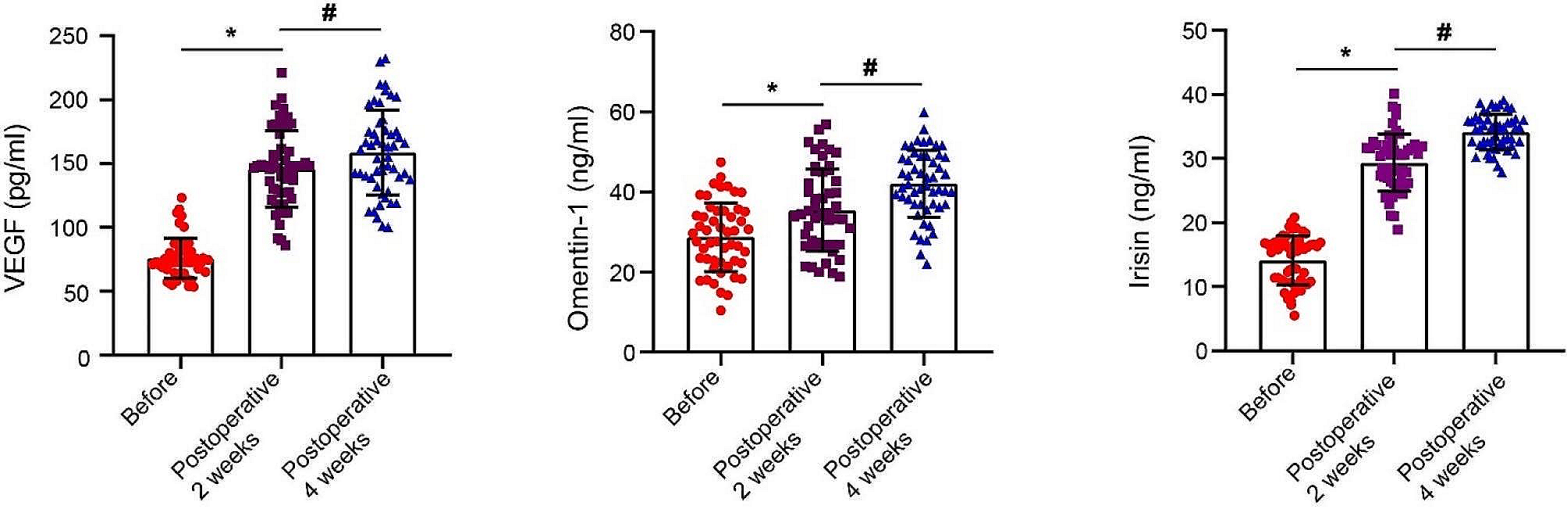
Dot plots showing serum levels of VEGF, omentin-1, and irisin in 52 DFU patients undergoing TTT on the preoperative 1st day, postoperative 2nd week and 4th week. p^*^ indicates significant difference when preoperative 1st day vs. postoperative 2nd weeks; p^#^ indicates significant difference when postoperative 2nd weeks vs. postoperative 4th weeks. The one-way ANOVA followed by Tukey’s post hoc test was performed for statistical analysis

The serum levels of VEGF, omentin-1, and irisin in DFU patients undergoing TTT were determined on the preoperative 1st day, postoperative 2nd week and 4th week. Results of ELISA methods showed the serum levels of VEGF, omentin-1, and irisin on the 2nd week and 4th week after TTT were notably elevated compared to the levels determined on the preoperative 1st day. The serum levels of VEGF, omentin-1, and irisin on the 4th week after TTT were also significantly higher than the levels determined on the 2nd week after TTT (Table 2; Fig. 6).

**Table 2:** The serum levels of VEGF, omentin-1, and irisin in DFU patients undergoing TTT on the preoperative 1st day, postoperative 2nd week and 4th week

Factor	Before	Postoperative 2nd weeks	Postoperative 4th weeks	*p* ^*^	*p* ^#^
VEGF (pg/ml, mean ± s.d.)	75.8 ± 15.5	145.9 ± 30.0	158.6 ± 33.1	< 0.0001	0.005
Omentin-1 (ng/ml, mean ± s.d.)	28.8 ± 8.6	35.5 ± 10.3	42.1 ± 8.3	< 0.0001	< 0.001
Irisin (ng/ml, mean ± s.d.)	14.2 ± 3.9	29.4 ± 4.5	34.2 ± 2.8	< 0.0001	< 0.0001

VEGF: vascular endothelial growth factor; p^*^ indicates preoperative 1st day vs. postoperative 2nd weeks; p^#^ indicates postoperative 2nd weeks vs. postoperative 4th weeks. The one-way ANOVA followed by Tukey’s post hoc test was performed for statistical analysis

## Discussion

In this prospective cohort study, TTT demonstrated its promising clinical benefits in the treatment of DFU. The treatment effect of this technique is associated with wound healing, limb salvage, and increased circulating levels of proangiogenic factors.

Previous surgical procedures for treating large diabetic foot lesions penetrating to the tendon, bone, or joint demonstrated a wound healing rate of 50-90% and a limb salvage rate of 50-94% during the 1-year follow-up, and a stable epithelization of 18-45% for at least 6 months [22]. In this study, we observed a wound healing rate of 92.3% and a limb salvage rate of 98.1% in patients with DFUs after TTT during the 1-year follow-up. We observed red granulation tissue gradually growing on the ulcers and the wound being much narrower with epithelization at the edges in the process of TTT. In most patients with severe DFUs, the wound was covered by robust, fresh granulation tissue at the postoperative 4th week and the ulcer was completely healed at the postoperative 12th week, indicating an earlier stable epithelization after TTT compared to previous surgical treatments. Sustained slow mechanically stretching by TTT stimulated blood vessel regeneration in the affected limb to rebuild microcirculation thus facilitating the healing of DFUs [23]. After distraction osteogenesis, the blood vessel volume density in the distracted callus and in the surrounding soft tissues on the movement side of the limb was increased, indicating distraction osteogenesis activated angiogenesis and maintained increasing vascularity [24]. An experimental study of TTT in rats also found the migration, proliferation, and angiogenesis of human umbilical vein endothelial cells were improved after TTT and thus the wound was ultimately accelerated [25]. El-Alfy et al. believed that distraction osteogenesis machinery could healed the soft tissue defects during the process of bone transport [26]. Choi et al. performed a scanning electron microscopic observation and found proliferating vessels of regenerating bone tissue, vascularization to the peripheral side of the interzone, and enhanced blood supply during distraction osteogenesis [27]. Concurring with our results, previous studies demonstrated TTT facilitated the ulcer healing, increased limb salvage, and improved health-related quality of life in patients with recalcitrant DFUs [28, 29]. In the study of heel ulcerations, TTT technique demonstrated its better achievement on wound healing [30]. Matsuyama et al. found that the distraction area exhibited more than three times greater ratio of average blood vessel volume than the intact contralateral tibiae [31], which was consistent with our results that TTT could significantly increase dorsal foot blood flow of patients at the 4th weeks after TTT. For better application of TTT in clinical practice, Liu et al. described the learning curve of surgeons performing TTT, demonstrating surgeons can master TTT after completing approximately 20 procedures and yielded almost consistent clinical outcomes in the initial implementation stages [32].

Earlier works have endeavored to dig out the mechanism explaining the treatment effect of TTT for DFUs, involving enhanced angiogenesis and inflammation modulation [33, 34]. In these works, the diabetic rat model with induced hindlimb ischemia presented marked neovascularization accompanying with upregulation of angiogenic factors, such as SDF-1 and CXCR4. VEGF is a well-recognized angiogenic factor, which was demonstrated to be increased in the serum sample of patients with DFU at the 4th week after TTT in our study, concurring with other studies [35, 36]. Most importantly, TTT has demonstrated its proangiogenic effects related to upregulation of omentin-1 and irisin in our study. Omentin-1 deficiency may contribute to delayed fracture healing and increased inflammation, accompanied by reduced production of platelet-derived growth factor-BB and osteogenesis-promoting vessels, indicating a positive role of omentin-1 in angiogenesis and inflammation modulation [37]. In the context of DFUs, administration of high-dose irisin was shown to restore high glucose-repressed migration and angiogenesis in human umbilical vein endothelial cell lines [38]. Irisin could alleviate inflammation, enhance vessel formation, and promote fracture repair [39].

There are several limitations needed to be taken into account for better interpretation of our data. First, this is a longitudinal study with a single-arm design and relatively small sample size, creating a caution to conclusion that TTT is better for the treatment of DFUs compare to previous surgical therapies. Second, the TTT procedures were conducted by a senior orthopaedic surgeon, creating a need of the learning curve of surgeons performing TTT in the initial stage compared to the mastery stage. Third, the patients were followed-up for 1 year in this study, and the wound healing, ulcer recurrence, and lower limb salvage at the 3-year or even 5-year follow-up should be analyzed.

In conclusion, our study demonstrates TTT as an alternative surgical method to effectively treat DFUs. TTT can promote the wound healing of the affected limbs, reduce lower limb amputation, and improve the blood circulation of the affected limbs. In the treatment of DFUs, significantly increased expressions of serum proangiogenic factors including VEGF, omentin-1, and irisin were noted in the early stage after TTT, which may provide a new mechanism of TTT promoting wound heal. Further clinical studies with two arms and large sample size during the prolonged follow-up period are required to validate the application of TTT in clinical practice of DFU treatment and limb salvage. Further preclinical studies are also performed to decipher the mechanism focusing on omentin-1 and irisin behind the treatment effect of TTT for DFUs.

## Data Availability

No datasets were generated or analysed during the current study.
